# The Impact of Repeat Endovascular Treatment on Critical Limb-Threatening Ischemia for Limb Salvage

**DOI:** 10.7759/cureus.59870

**Published:** 2024-05-08

**Authors:** Masanori Takada, Takao Maruyama

**Affiliations:** 1 Cardiology, Medical Corporation Kawasaki Hospital, Kobe, JPN

**Keywords:** chronic limb-threatening ischemia, bypass surgery, limb salvage, repeat endovascular treatment, below-the-knee stenting

## Abstract

Chronic limb-threatening ischemia due to chronic total occlusion of below-the-knee lesions is one of the most challenging cases for endovascular treatment. Restoring perfusion is crucial, and its success depends on numerous factors. Owing to the recent development of dedicated devices and techniques, endovascular treatment is becoming an alternative to bypass surgery as a first-line treatment, even for the infra-popliteal lesion, because endovascular recanalization outcomes have considerably improved. In our present case, a self-expandable Nitinol stent was placed in the tibio-peroneal trunk to treat chronic limb-threatening ischemia. At its recurrence four years later, endovascular therapy was employed because the patient had concomitant diseases and advanced age. Finally, four times repeated revascularization prevented major amputation and preserved the functional foot. This report demonstrates that repeated endovascular therapy was practical and feasible to achieve limb salvage and preserve the functional foot.

## Introduction

Chronic limb-threatening ischemia (CLTI) is the most advanced stage of atherosclerotic disease of the lower extremities. Bypass surgery is an effective and durable revascularization strategy for CLTI; however, patients with CLTI often have multiple comorbidities and do not always undergo bypass surgery. On the other hand, endovascular therapy (EVT) is becoming a viable option because of its reproducibility and low invasiveness [1]. Despite the disadvantage of a high reintervention rate, it has the advantage of repeatable re-treatment; the goal of revascularization in CLTI is limb salvage through wound healing and to prevent major amputation. So, stent implantation in below-the-knee (BTK) lesions might be effective in restoring perfusion of complex lesions [2], but it is controversial.

Moreover, concerning revascularization for recurrent CLTI, EVT may have a worse prognosis than surgical revascularization [3], but which procedure should be taken depends on the case. Generally, endovascular revascularization for recurrence is extremely difficult, with multidisciplinary treatment and skilled repeat EVT, in which invasive treatment was avoided, and lower extremity function was preserved. In this case report, we demonstrate the safety and efficacy of repeated EVT for recurrent CLTI.

## Case presentation

A man in his 80s on hemodialysis was hospitalized (Rutherford category 5) for resting pain and swelling on the dorsal side of the left foot four months after he had undergone left first-digit amputation due to a diabetic ulcer. He had a history of coronary artery disease, lower extremity artery disease (LEAD), and a trans-metatarsal amputation (TMA) of the right foot due to diabetic ulcer. EVT had been performed to treat occluded tibioperoneal trunk (TPT) caused by CLTI; a self-expandable nitinol SMART stent (Cordis, USA) was placed in the TPT for bail-out procedure four years back.

**Figure 1:**
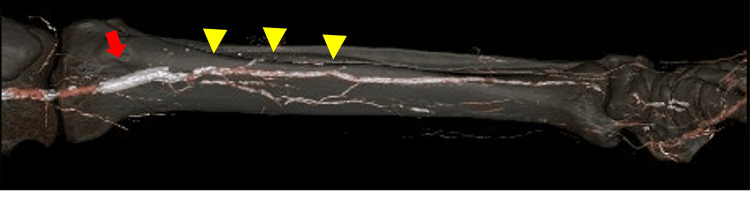
Contrast-enhanced computed tomography (CECT) image Both the anterior tibial artery (ATA) and the posterior tibial artery (PTA) were jailed by the SMART stent (red arrow) and the peroneal artery (yellow arrowhead) could be only detected.

**Figure 2:**
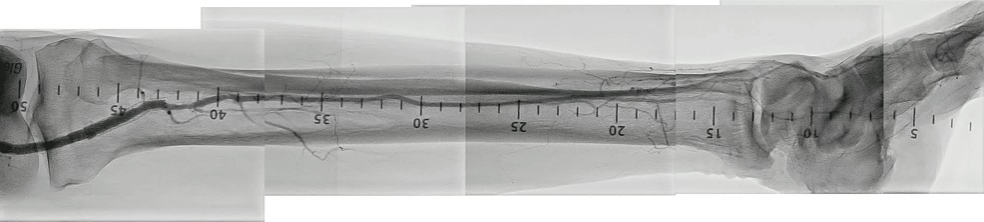
The diagnostic angiography of the first attempt.

The patient underwent imaging tests to plan the recanalization strategy. Magnetic resonance T2 weighed imaging revealed high intensity around the left second digit, suggesting osteomyelitis. Contrast-enhanced computed tomography (CECT) images revealed that both the anterior tibial artery (ATA) and the posterior tibial artery (PTA) were jailed by the SMART stent, and the peroneal artery could be only detected (Figure 1). Physiologic testing revealed skin perfusion pressure (SPP) of 20 mmHg on the dorsal surfaces of the left foot. As a result, in the present case, the clinical stage was four by WIfI classification: Wound 2, Infection 3, and foot Infection 1. After a multidisciplinary team discussed the patient’s condition, we chose percutaneous recanalization over bypass surgery to obtain direct ATA flow to the dorsal artery of the left foot immediately after second digit debridement. The diagnostic angiography from the popliteal artery revealed findings similar to those of CECT (Figure 2). Because the entry of the ATA was obscured due to the SMART stent, the retrograde approach from the dorsal artery was decided. After the 0.014-inch floppy guidewire supported by the microcatheter was inserted retrogradely into the pedal artery and up to the proximal occlusion site in the ATA. The retrograde wire with microcatheter was peripherally attached to the vessel outside the stent. The intravascular ultrasound (IVUS) findings in the stent showed severe calcified and dense plaque. While failing to penetrate the vessel wall despite using the hard 50g wire, the wire-tail pecking was decided.

**Figure 3:**
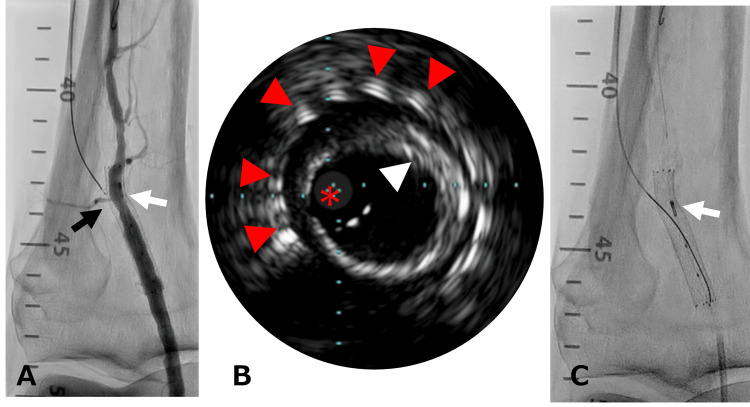
White arrow; IVUS. (A) The retrograde wire and microcatheter (black arrow). (B) IVUS image: SMART stent (red arrowhead), dense calcified plaque (white arrowhead), IVUS image core (asterisk). (C) Wire-tail penetration into the stent. IVUS = intravascular ultrasound

**Figure 4:**
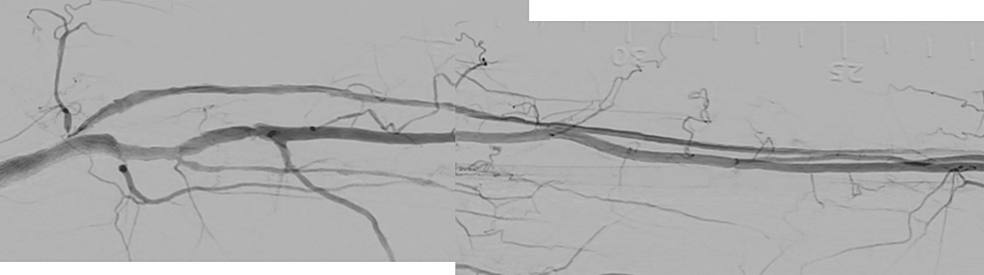
The final angiography of the first attempt.

Finally, we could advance the wire into the stent (Figure 3). Then, the retrograde guidewire with microcatheter was introduced into the antegrade sheath and externalized successfully to make it pull through. Subsequently, we dilated the CTO lesion and SMART stent strut with a 2.0-mm balloon. The final angiography was optimal and revealed direct ATA flow to the ischemic lesion (Figure 4).

**Figure 5:**
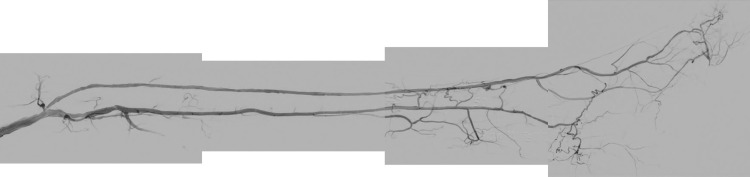
The final angiography after the fourth EVT.

The left foot wound had incomplete healing despite deliberate care by the interventional cardiologists, plastic surgeons, and paramedical staff. Two months after the final EVT, the dorsal area declined in SPP, and a second attempt of the left BTK lesion was decided. Angiography revealed the expected ATA occlusion due to the vascular recoil phenomenon. Repeat EVT was performed in the antegrade fashion without difficulty. A 2.5 mm balloon was dilated in the occlusion site, and the final angiography revealed that the direct inflow to the dorsal lesion had improved. Two months after the second attempt, left TMA was performed to avoid major amputation due to a worsening infectious wound. Angiography was performed to determine perfusion to the anastomosis; this revealed the slow-flow phenomenon due to ostial ATA stenosis without occlusion. Another repeat EVT was done and the ATA was dilated with a 2.5 mm balloon. The final angiography revealed improved flow. After this third attempt, the patient’s left lower extremity condition improved with continuous multidisciplinary care. Six months after the first attempt, we performed follow-up angiography due to residual skin coldness of the left foot despite 46 mmHg SPP on the dorsal surface. Angiography revealed the antegrade slow-flow phenomenon. We successfully performed a fourth EVT for ATA to avoid CLTI recurrence. The final angiography revealed an optimal result (Figure 5). From then until this report, he remained an outpatient without any symptoms or need for equipment for his foot.

## Discussion

CLTI has a poor prognosis associated with resting pain and ulceration/gangrene due to chronic ischemia, and the essential treatment goals are wound healing and limb salvage. Therefore, revascularization is mandatory to ameliorate ischemia. In clinical settings, bypass surgery has been the gold standard for revascularization in CLTI patients. However, since the BASIL trial [4], EVT has become a first-line treatment for CLTI patients with perioperative risks or comorbidities. Although it has the disadvantages of high restenosis and reintervention rates, it also has the advantages of repeating the procedure until wound healing and limb salvage are achieved, the low cost and short hospital stay [5], and the remarkable technological advances and instrumentation developments.

The SPINACH registry suggested that CLTI patients with severe limb conditions would be suitable for bypass surgery. In contrast, patients in poor general conditions, such as frailty, have also been shown to benefit more from EVT [3]. Considering the criteria of this registry, we found that both EVT and bypass surgery could be options for the present case. On the other hand, some reports suggest that patients with multivessel disease should undergo endovascular revascularization rather than surgical reconstruction [4]. As for wound healing, Bakal et al. demonstrated the importance of restoring blood flow to the leg in a straight line [6]; the most critical EVT endpoint for CLTI is to ensure adequate blood flow for wound healing and, in some cases, to achieve a straight line, taking the angiosome into account [7]. Peri-wound SPP is the most crucial circulatory assessment tool for wound healing. Especially in hemodialysis patients, SPP is frequently used to detect LEAD and has better sensitivity and specificity than other indicators such as ankle-brachial index (ABI), toe brachial index (TBI), and transcutaneous oxygenation measurement (TcPO2) [8]. To achieve wound healing, SPP must be greater than 30 mmHg. In the present case, SPP was used to determine whether ischemia in the treated lesion had progressed postoperatively and the re-intervention time.

The present case had a recurrent CLTI concomitant with osteomyelitis, and it was challenging to achieve limb salvage. Although the safety and efficacy of the retrograde approach for CLTI have been reported [9,10], to our knowledge, this is the first report of revascularization of a CLTI caused by a completely occluded lesion beyond the stent strut. Meanwhile, there was concern that the stent strut was not fully dilated after balloon dilation in this procedure. Unlike coronary stents, balloon dilation could be ineffective due to the relevant characteristics of self-expandable stents with micromesh cell design. Therefore, residual stenosis was often observed on the angiography. Nevertheless, the primary goal of EVT for CLTI is limb salvage, not the appearance of a larger conduit. It is essential to obtain direct flow to the ischemic lesion along the angiosome [11].

A recurrence of CLTI complicated by infection was imminent for limb salvage, but repeat EVT, as in the case, could avoid the major amputation. The timing of re-treatment was determined regarding the SPP value, and the direct in-flow to the wound lesion at each final angiogram allowed time for wound healing and salvage. Currently, the number of elderly hemodialysis patients with diabetes is rapidly increasing. Percutaneous revascularization for CLTI has made remarkable progress, and EVT should be considered a first attempt and repeated for complex lesions.

## Conclusions

The present case illustrates that, for CLTI with multiple comorbidities and a high risk of amputations, repeat EVT with skilled technique enabled the gain of time for wound healing. It could avoid major amputations and improve the patient’s daily life, even if the recurrence status.
